# Colombian registry of inflammatory bowel disease in pediatrics

**DOI:** 10.1093/crocol/otag038

**Published:** 2026-08-31

**Authors:** Fernando Sarmiento Quintero, Ana María Castañeda Figueroa, Diana Victoria Mora Quintero, Claudia Patricia Sánchez Franco, Lina Eugenia Jaramillo Barberi, Sergio Andrés Prado Baquero, Marco Alberto Suárez Urueña, Andrea Natalia Pedraza Peña, Stephanía Peña Hernández, Karol Andrea Martínez Portilla, Lina Baños Rocha, Luz Esthella Tovar Correa, Diana Catalina López Mora, Catalina Pallejá López, Diana Paola Sánchez Hernández, Mónica María Contreras Ramírez, Catalina Ortiz Piedrahita, Claudia Liliana Losada Gómez, Alejandra Wilches Luna, Diana María Quimbayo Wilches, Verónica Botero Osorio, Rafael Milanés Romero, Andrés Enríquez Calvache, Melquisedec Vargas Sandoval, Fernando Medina Monroy, Hugo Laignelet Hernández, Wilson Daza Carreño, José Fernando Vera Chamorro, Ailim Margarita Carias Domínguez, Johana Hincapié Butto, Maira Patricia Sánchez Pérez, Carlos Cuadros Mendoza, Otto Calderón Guerrero, María Mercedes Naranjo Vergara, Clara Eugenia Plata García, Luis Carlos Ramírez Urrego, Rodrigo De Vivero Camacho, Sandra Helena Paipilla Monroy, Luz Eugenia Aragón Calvo, Mariastella Serrano

**Affiliations:** Pediatric Gastroenterology Unit, Department of Pediatrics, Universidad Nacional de Colombia, Bogotá, Colombia; Paediatric Gastroenterology Unit, HOMI Fundación Hospital La Misericordia, Bogotá, Colombia; Epidemiology Unit, Universidad Nacional de Colombia, Bogotá, Colombia; Paediatric Gastroenterology Unit, HOMI Fundación Hospital La Misericordia, Bogotá, Colombia; Paediatric Gastroenterology Unit, HOMI Fundación Hospital La Misericordia, Bogotá, Colombia; Pediatric Gastroenterology Unit, LaCardio Fundacion Cardioinfantil Instituto de Cardiologia, Bogotá, Colombia; Paediatric Gastroenterology Unit, HOMI Fundación Hospital La Misericordia, Bogotá, Colombia; Pathology Unit, Universidad Nacional de Colombia, Bogotá, Colombia; Epidemiology Unit, Universidad Nacional de Colombia, Bogotá, Colombia; Paediatric Gastroenterology Unit, HOMI Fundación Hospital La Misericordia, Bogotá, Colombia; Paediatric Gastroenterology Unit, HOMI Fundación Hospital La Misericordia, Bogotá, Colombia; Paediatric Gastroenterology Unit, HOMI Fundación Hospital La Misericordia, Bogotá, Colombia; Paediatric Gastroenterology Unit, HOMI Fundación Hospital La Misericordia, Bogotá, Colombia; Paediatric Gastroenterology Unit, HOMI Fundación Hospital La Misericordia, Bogotá, Colombia; Paediatric Gastroenterology Unit, HOMI Fundación Hospital La Misericordia, Bogotá, Colombia; Pediatric Gastroenterology Unit, Department of Pediatrics, Universidad Nacional de Colombia, Bogotá, Colombia; Pediatric Gastroenterology Unit, Department of Pediatrics, Universidad Nacional de Colombia, Bogotá, Colombia; Pediatric Gastroenterology Unit, Hospital Pablo Tobón Uribe, Medellín, Colombia; Pediatric Gastroenterology Unit, Hospital Pablo Tobón Uribe, Medellín, Colombia; Pediatric Gastroenterology Unit, Hospital Pablo Tobón Uribe, Medellín, Colombia; Pediatric Gastroenterology Unit, Hospital San Vicente de Paul, Medellín, Colombia; Pediatric Gastroenterology Unit, Hospital San Vicente de Paul, Medellín, Colombia; Pediatric Gastroenterology Unit, Lili Valley Foundation, Cali, Colombia; Pediatric Gastroenterology Unit, Lili Valley Foundation, Cali, Colombia; Pediatric Gastroenterology Unit, Lili Valley Foundation, Cali, Colombia; Pediatric Unit, Universidad Tecnológica de Pereira Facultad de Ciencias de la Salud, Pereira, Colombia; Pediatric Unit, Universidad Tecnológica de Pereira Facultad de Ciencias de la Salud, Pereira, Colombia; Pediatric Unit, Universidad Industrial de Santander, Bucaramanga, Colombia; Pediatric Unit, Hospital Militar Central, Bogotá, Colombia; Nestlé Nutrition Institute, Bogotá, Colombia; Pediatric Gastroenterology Unit, Fundación Santa Fé de Bogotá, Bogotá, Colombia; Pediatric Gastroenterology Unit, Fundación Santa Fé de Bogotá, Bogotá, Colombia; Paediatric Gastroenterology Unit, Hospital Universitario del Valle Evaristo Garcia, Cali, Colombia; Paediatric Gastroenterology Unit, Hospital Universitario del Valle Evaristo Garcia, Cali, Colombia; Pediatric Gastroenterology Unit, Fundación Cardiovascular de Colombia, Bucaramanga, Colombia; Pediatric Gastroenterology Unit, Centro Médico Imbanaco, Cali, Colombia; Pediatric Gastroenterology Unit, Centro Médico Imbanaco, Cali, Colombia; Pediatric Gastroenterology Unit, Pontificia Universidad Javeriana, Bogotá, Colombia; Pediatric Unit, Hospital Federico Lleras Acosta, Ibagué, Colombia; Paediatric Gastroenterology Unit, Universidad de Cartagena Facultad de Medicina, Cartagena, Colombia; Pediatric Unit, Fundación Universitaria de Ciencias de la Salud, Bogotá, Colombia; Pediatric Gastroenterology Unit, Centro Médico Imbanaco, Cali, Colombia; Pediatric Gastroenterology, Hepatology and Nutrition, MedStar Georgetown University Hospital, Washington, D.C., United States

**Keywords:** registry, inflammatory bowel disease, pediatrics

## Abstract

**Background:**

Inflammatory bowel disease (IBD) has significantly increased over the past two decades. However, registries in Latin America remain scarce, prompting the creation of the Colombian pediatric IBD registry for patients under 18 years of age, designed as a continuous and periodically assessed database.

**Methods:**

A descriptive, longitudinal, ambispective, and multicenter observational study was conducted in children aged 0-18 years between January 1999 and December 2021. The analysis was performed based on the age of onset (Paris classification), with subdivisions for very early onset and phenotypes using the Pediatric IBD-classes algorithm. Data were collected in REDCap and analyzed with STATA 15.1.

**Results:**

A total of 209 patients were included, 54.5% male. The median age at diagnosis was 12.3 years (IQR 8.5-14.6). Phenotype distribution included: 104 (53.06%) typical ulcerative colitis (TUC), 52 (26.53%) Crohn’s disease (CD), 18 (9.18%) unclassifiable IBD (U-IBD), 11 (5.61%) colonic Crohn’s disease (CCD), and 11 (5.61%) atypical ulcerative colitis (AUC). The median time to diagnosis was 6.6 months, and treatment began after a median of 11.2 days. Coffee-cultivation zone 39%. Extraintestinal manifestations were reported in 43.9% of cases. Initial treatments included mesalamine ± steroids (35.86%), immunomodulators ± steroids (32%), exclusive/partial enteral nutrition (11.41%), and biologics ± immunomodulators (20.65%), which increased to 57.7% during follow-up. Relapse occurred in 53.26% of patients, while 35.32% achieved remission.

**Conclusions:**

This registry highlights the increasing incidence of pediatric IBD in Latin America, providing valuable insights into clinical presentations, diagnostic delays, treatment strategies, and outcomes, while revealing regional and global disparities.

## Introduction

Inflammatory bowel disease (IBD) is a chronic autoimmune disorder that encompasses Crohn’s disease (CD) and ulcerative colitis (UC) with no known cause, with gastrointestinal involvement and extraintestinal manifestations (EIM).^1^^,^^2^ These conditions significantly impact patients’ quality of life,^3^ and show an increasing global prevalence in children and adults.^4^^,^^5^ Similar trends have been observed in Latin America^6^ and Colombia^7^ in both children and adult, but there are scarce detailed reports on these entities in these countries. In Colombia, the first pediatric case of UC was documented in 1999 in a 14-year-old adolescent at a pediatric hospital in Bogotá (personal communication). Thereafter the cases increased gradually and by 2012, in that same hospital there was one case diagnosed monthly (personal communication). During a national survey conducted in 2013, 66 pediatric cases were reported (36 with UC, 27 with CD and 3 with indeterminate IBD), (personal communication). The same situation has been observed in adult patients: 108 cases had been reported (98 UC and 10 CD) between 1968 and 1990,^8^ this number had risen to 2291 cases (1813 UC, 456 CE and 22 non-classifiable IBD) by 2021.^9^

Unclassifiable IBD, (U-IBD), is observed in a small percentage of patients and has led to the development of a classification system of IBD, that includes age of onset and phenotype. Current classification based on age, divided it into early-onset in children under 10 and pediatric under 17 years old.^10^ For children diagnosed before the age of 10, the classification further subdivides into: neonatal onset NOIBD (before 28 days), infantile-onset IOIBD (before 24 months), and very early-onset VEOIBD (before 6 years).^11^ Furthermore, the pediatric IBD (PIBD) classes classification system identifies 5 phenotypes: typical ulcerative colitis (TUC), Crohn’s disease (CD), unclassifiable IBD (U-IBD), colonic Crohn’s disease (CCD), and atypical ulcerative colitis (AUC).^12^^,^^13^

In Latin America, there are no registries that demonstrate the behavior of the disease as has been documented in high-income countries^14^^,^^15^; This is the rationale for initiating the Colombian registry of IBD in children under 18 years of age which will be continuous and with periodic assessments, with classification by age of onset and by phenotype. The registry encompasses demographic information, clinical presentation, diagnostic parameters, time to diagnosis, treatment, management, and outcomes. It also highlights the differences between this population and populations in other parts of the world.

## Methods

This observational, descriptive, longitudinal, ambispective, and multicenter study included 209 patients aged 0 and 18 years old with IBD diagnosed from January 1, 1999, to December 31, 2021. Patients were followed until loss of follow-up, transition to adult gastroenterology care or death, by 34 pediatric gastroenterologists from 17 institutions and 5 private practices in 9 Colombian cities. All variables were recorded in the REDCap database. Ethical approval was obtained from the institutional review boards of all the participating institutions, and the identity of the patients was concealed under an alphanumeric code known only to two of the researchers in accordance Statutory Law of Habeas Data and the Declaration of Helsinki.

Diagnosis was determined according to age of onset^10^^,^^11^ and validated PIBD-class algorithm^12^^,^^13^ in 196 of the 209 patients by review of clinical records and supporting studies completed in these patients analyzed retrospectively.

Since this new classification was described only in 2017 and not all Colombian pediatric gastroenterologists are familiar with it, the IBD-classes Catherine McEwan Foundation App and a manual instruction were made available in order to reclassify all patients diagnosed before 2017 or the ones that needed to be reclassified even after 2017. To minimize bias, the following mechanisms were implemented: (1) integration of the IBD Classes Catherine McEwan Foundation App into REDCap, (2) verification and clarification of inconsistent data, (3) review and correction of digitalized variables values, and (4) confirmation of the adequate phenotypic classification of the patients by one of the investigators, with previous training in its application.

Data were imported from REDCap to a comma-separated values format and analyzed using Stata 15.1. Descriptive statistics were used for numerical variables, and measures of central tendency (median) and dispersion (interquartile ranges [IQR]) were used. Qualitative variables were presented as absolute and relative frequencies. Analyses were stratified by age of symptom onset and phenotype based on the “PIBD-classes classification.” Comparative studies of stratified characteristics were conducted using statistical tests including the Mann-Whitney U test, sign ranking test for paired data, Kruskal-Wallis test, Chi-square test, McNemar test or Fisher’s exact test, establishing a statistical significance of *P* ≤ .05.

## Results

### Sociodemographic characteristics

Among the total cohort, 209 patients, 54.5% were boys; the median age was 12.3 years (IQR 8.5-14.6), ranging from 25 days to 19.5 years. Comprehensive phenotype and age-of-onset data were available for 196 patients, who were stratified based on these variables (Table 1).

**Table 1:** Distribution of patients by phenotypes and age of onset.

N = 196 (%)	TUC	CD	U-IBD	CCD	AUC	Total
**Neonatal**	0 (0.00)	0 (0.00)	1 (0.51)	0 (0.00)	0 (0.00)	1 (0.51)
**Infantile**	5 (2.56)	3 (1.54)	1 (0.51)	0 (0.00)	0 (0.00)	9 (4.59)
**Very early onset**	5 (2.56)	3 (1.54)	1 (0.51)	1 (0.51)	1 (0.51)	15 (7.65)
**Early onset**	26 (13.33)	10 (5.13)	4 (2.05)	3 (1.54)	5 (2.56)	44 (22.45)
**Pediatrics**	68 (34.69)	36 (18.46)	11 (5.64)	7 (3.59)	5 (2.56)	127 (64.79)
**Total**	**104 (53.06)**	**52 (26.53)**	**18 (9.18)**	**11 (5.61)**	**11 (5.61)**	**196** ^a^

a Complete data on phenotype and age of onset available for 196 patients. Summarizes the characteristics of the cohort: in the rows the distribution by age of onset and in the columns by phenotype.

Abbreviations: AUC, atypical ulcerative colitis; CCD, colonic Crohn’s disease; CD, Crohn’s disease; TUC, typical ulcerative colitis; U-IBD, unclassifiable inflammatory bowel disease.

Prevalence rates (cases/1000 patients/year) were: TUC 22.6, CD 11.3, U-IBD 3.9, AUC 2.4 and CCD 2.4.

**Figure 1:**
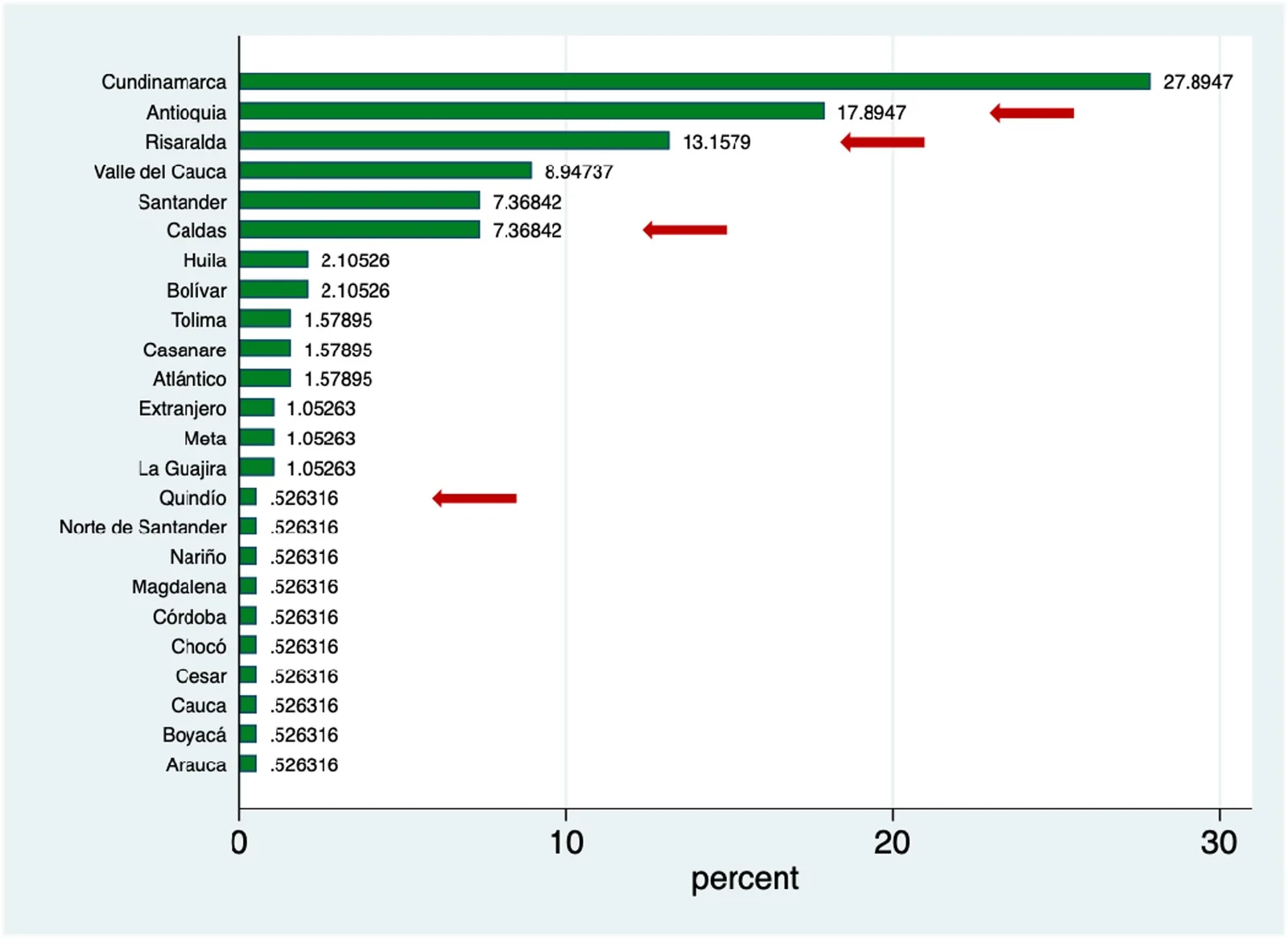
Incidence of IBD cases based on patients’ province. The arrows indicate the 4 predominant areas with coffee crops and similar environmental conditions and sea level altitude. Characteristics also observed in IBD in adults.^12^ The sum far exceeds the percentage of the province with the highest number of cases.

Of the total, 177 patients came from the urban areas, and 39% from the Andean region, a predominantly coffee-cultivation zone. As illustrated in Figure 1, the combined prevalence rates in 4 coffee-growing provinces surpassed those in non-coffee-growing areas.

Out of the 118 patients with documented socioeconomic status, 98 individuals (83%) were categorized within the 3 lowest socioeconomic strata. Specifically, 13 patients belonged to stratum 1, 38 to stratum 2, and 47 to stratum 3.

Patient care was predominantly provided in hospital institutions affiliated with medical schools or universities, accounting for 69% of cases. Conversely, only 29% of patients received care in private practices without academic affiliation.

### Clinical features

**Figure 2:**
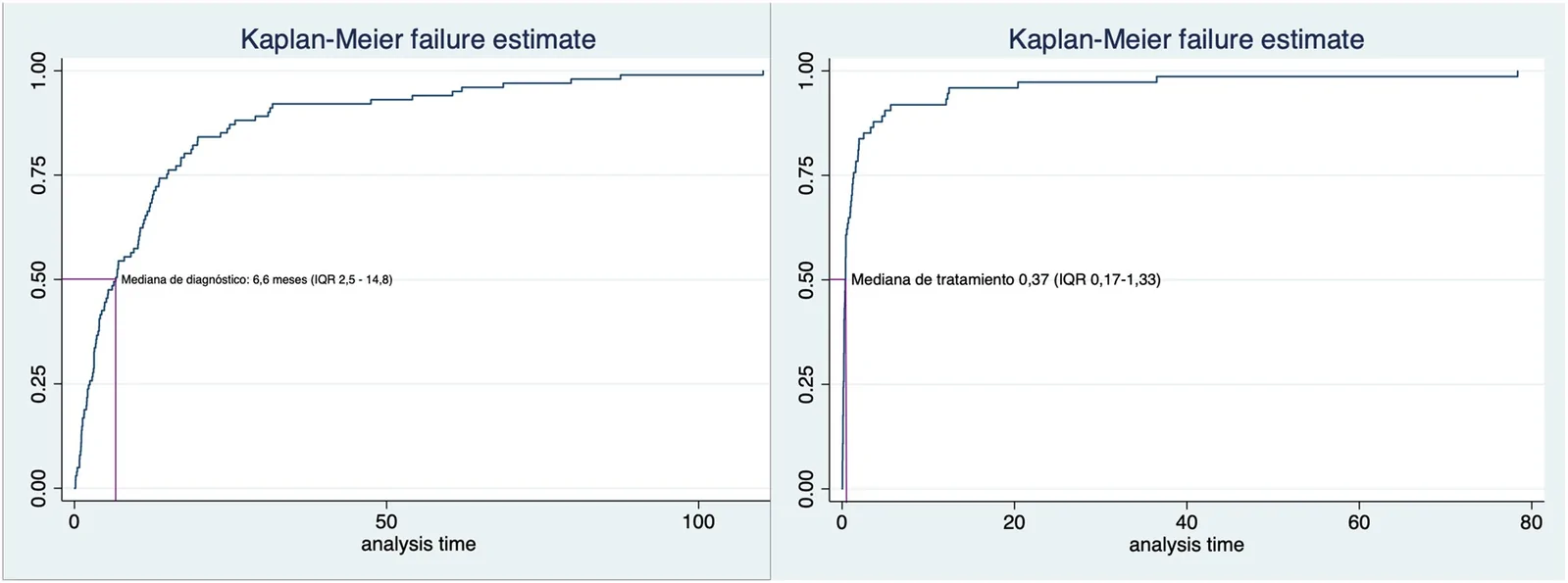
Time elapsed between onset of symptoms to diagnosis (A) and the initiation of treatment (B).

In 101 patients in whom complete data of age of onset of symptoms and time of diagnosis were available, the median time from symptom onset to diagnosis was 6.6 months (IQR 2.5-14.8), ranging from 5.1 days to 11.2 years. Graph 3. Data from 74 patients in whom details of the time of diagnosis and time of initiation of treatment were available, reveals that the median from time of diagnosis to initiation of treatment was 11.2 days (IQR 5.2-40 days), ranging from 1 day to a maximum of 6.5 years (Figure 2).

Table 2 describes the signs and symptoms according to phenotype. Diarrhea, abdominal pain, rectal bleeding, and nocturnal bowel movements were predominant in TUC, whereas fever of unknown cause and vomiting were more commonly associated with CD. Anal fistulas and skin-tags were exclusive in CD and CCD.

**Table 2:** Signs and symptoms per phenotypes.

N = 196	TUC *N *= 104	CD *N *= 52	U-IBD *N *= 18	CCD *N *= 11	AUC *N *= 11	*P* value^a^
**Diarrhea**	94 (47.9)	40 (20.4)	13 (6.6)	9 (4.6)	10 (5.1)	.078
**Abdominal pain**	91 (48.6)	46 (24.6)	11 (5.8)	9 (4.8)	7 (3.7)	.028
**Rectal bleeding**	98 (51.5)	33 (17.3)	15 (7.9)	8 (4.2)	9 (4.7)	<.001
**Nocturnal bowel movements**	56 (32.5)	18 (10.4)	5 (2.9)	5 (2.9)	2 (1.1)	.057
**Interruption of activities**	45 (25.5)	18 (10.2)	5 (2.8)	4 (2.2)	4 (2.2)	.920
**Weight loss**	50 (27.4)	36 (19.7)	10 (5.4)	7 (3.8)	5 (2.7)	.164
**Growth failure**	18 (10.3)	15 (8.6)	5 (2.8)	5 (2.8)	1 (0.5)	.114
**Vomiting**	10 (5.6)	13 (7.4)	4 (2.2)	5 (2.8)	3 (1.7)	.004
**Fever with unknown source**	12 (6.8)	15 (8.5)	0 (0.0)	2 (1.1)	0 (0.0)	.012
**Oral thrush**	11 (6.3)	11 (6.2)	1 (0.5)	2 (1.1)	0 (0.0)	.238
**Peripheral arthropathy**	15 (8.5)	8 (4.5)	0 (0.00)	2 (1.1)	1 (0.5)	.624
**Anal fissure**	5 (2.8)	6 (3.4)	1 (0.5)	3 (1.7)	0 (0.0)	.084
**Puberty delay**	5 (3.0)	7 (4.2)	0 (0.0)	2 (1.2)	0 (0.0)	.097
**Erythema nodosum**	6 (3.5)	6 (3.5)	0 (0.0)	1 (0.6)	0 (0.0)	.470
**Skin-tags**	0 (0.00)	7 (4.0)	0 (0.0)	1 (0.5)	0(0.0)	.001
**Central arthropathy**	5 (2.8)	2 (1.1)	0 (0.00)	2 (1.1)	0 (0.0)	.367
**Perianal fistula**	0 (0.0)	7 (3.9)	0 (0.0)	0 (0.0)	0 (0.0)	.002
**Hepatitis**	4 (2.2)	1 (0.5)	0 (0.0)	1 (0.5)	0 (0.0)	.662
**Sclerosing cholangitis**	2 (1.1)	1 (0.5)	0 (0.0)	1 (0.5)	0 (0.0)	.549
**Osteopenia**	1 (0.6)	1 (0.6)	0 (0.0)	1 (0.6)	0 (0.0)	.433
**Pyoderma gangrenosum**	0 (0.00)	3 (1.7)	0 (0.0)	0 (0.0)	0 (0.0)	.149
**Uveitis**	1 (0.6)	0 (0.00)	0 (0.0)	1 (0.6)	0 (0.0)	.260
**Scleritis**	1 (0.6)	1 (0.6)	0 (0.0)	0 (0.0)	0 (0.0)	1.000

The frequency of the first 3 signs and symptoms is similar to that reported in the literature, although diarrhea is not significant. Abdominal pain, vomiting, and fever of unknown origin point significantly to TUC and CD, and rectal bleeding is a capital sign. On the other hand, perineal findings were only presented in CD and CCD.

a Fisher’s exact test.

Abbreviations: AUC, atypical ulcerative colitis; CCD, colonic Crohn’s disease; CD, Crohn’s disease; TUC, typical ulcerative colitis; U-IBD, unclassifiable inflammatory bowel disease.

Of 196 patients analyzed, 86 (43.9%) had extraintestinal manifestations (EIM). Breaking down by phenotype, EIM prevalence was highest in CCD (63.63%; 7/11), followed by CD (55.76%; 29/52), TUC (40.38%; 42/104), U-IBD (27.77%; 5/18), AUC (18.2%; 2/11). Of 86 patients with EIM, 57 (66.28%) with one manifestation; with two 16 (18.60%); and three or more 13 (15.2%). Overall, EIM were less frequent in children under 6 years of age (Table 3).

**Table 3:** EIM presence per phenotype and age of onset.

Patients 86/196 (43.9%)	CCD *N* = 7/11	CD *N* = 29/52	TUC *N* = 42/104	U-IBD *N* = 5/18	AUC *N* = 2/11	Child *N* = 4	Very early *N* = 4	Early *N* = 21	Pediatrics *N* = 56
**Growth failure**	5 (71.4)	15 (51.7)	18 (42.8)	5 (100)	1 (50.0)	2 (50.0)	4 (100)	14 (66.6)	24 (42.8)
**Peripheral arthropathy**	2 (28.5)	8 (27.6)	15 (35.7)	0	1 (50.0)	1 (25.0)	0	6 (28.5)	19 (33.9)
**Oral thrush**	2 (28.5)	11 (37.9)	11 (26.2)	1 (20.0)	0	1 (25.0)	2 (50.0)^a^	5 (23.8)	17 (30.3)
**Puberty delay**	2 (28.5)	7 (24.1)	5 (11.9)	0	0	0	0	4 (19.0)	10 (17.8)
**Erythema nodosum**	1 (14.2)	6 (20.7)	6 (14.3)	0	0	0	0	0	13 (23.2)
**Central arthropathy**	2 (28.5)	2 (6.9)	5 (11.9)	0	0	0	1 (25.0)^a^	2 (9.5)	6 (10.7)
**Skin tags**	1 (14.2)	7 (24.1)	0	0	0	0	1 (25.0)^a^	1 (4.7)	6 (10.7)
**Hepatitis**	1 (14.2)	1 (3.4)	4 (9.5)	0	0	0	0	2 (9.5)	4 (7.1)
**Sclerosing cholangitis**	1 (14.2)	1 (3.4)	2 (4.7)	0	0	0	0	0	4 (7.1)
**Osteopenia**	1 (14.2)	1 (3.4)	1 (2.4)	0	0	0	1 (25.0)^a^	1 (4.7)	1 (1.8)
**Pyoderma gangrenosum**	0	3 (10.3)	0	0	0	0	0	1 (4.7)	2 (3.5)
**Uveitis**	1 (14.2)	0	1 (2.4)	0	0	0	0	0	2 (3.5)
**Scleritis**	0	0	1 (2.4)	0	0	0	0	0	1 (1.8)
**With one manifestation 57 patients (66.28%), with two 16 (18.60%), and with three or more 13 (15.2%)**									

a This table also shows the distribution by age of onset in which oral thrush, central arthropathy, skin tags, and osteopenia are observed in children under 2 years of age. No EIMs occurred in children younger than 28 days.

Abbreviations: AUC, atypical ulcerative colitis; CCD, colonic Crohn’s disease; CD, Crohn’s disease; TUC, typical ulcerative colitis; U-IBD, unclassifiable inflammatory bowel disease.

A total of 135 patients (64.59%) required in-hospital management, and Table 4 shows the laboratory results according to phenotype, which show that hemoglobin of less than 10 g% was only present in less than 25% of patients. Anti-saccharomyces cerevisiae antibodies (ASCA) were positive more often in patients with CD with statistical significance. Finally, we noted that only a low percentage of patients had positive stool cultures and/or positive *Clostridiodes Difficile* assays.

**Table 4:** Laboratory characteristics according to phenotype.

Exam (# of patients included in analysis)	Ranges	Total (%)	TUC *N *= 104 (%)	CD *N *= 52 (%)	IBDU *N *= 18 (%)	CCD *N *= 11 (%)	AUC *N *= 11 (%)	*P* value
**Hemoglobin gr/dL** **180 patients**	<7 7-10 >10	7 (3.89) 39 (21.67) 134 (74.44)	7 (3.89) 19 (10.56) 73 (40.56)	0 (0.00) 15 (8.33) 34 (18.89)	0 (0.00) 2 (1.11) 10 (5.56)	0 (0.00) 2 (1.11) 9 (5.00)	0 (0.00) 1 (0.56) 8 (4.44)	.496^a^
**Platelets^b^** **174 patients**	<100 100-500 >500	39 (22.41) 85 (48.85) 50 (28.74)	28 (16.09) 45 (25.86) 22 (12.64)	4 (2.30) 25 (14.37) 19 (10.92)	0 (0.00) 7 (4.02) 4 (2.30)	3 (1.72) 5 (2.87) 3 (1.72)	4 (2.30) 3 (1.72) 2 (1.15)	**.027^a^**
**CRP** **mg/dL** **159 patients**	<6 6-20 >20	98 (61.64) 24 (15.09) 37 (23.27)	63 (39.62) 10 (6.29) 15 (9.43)	15 (9.43) 11 (6.92) 19 (11.9)	7 (4.40) 1 (0.63) 2 (1.26)	9 (5.66) 1 (0.63) 0 (0.00)	4 (2.52) 1 (0.63) 1 (0.63)	**.001^a^**
**GOT U/L** **151 patients**	Median (IQR)	20 (16.0- 29.0)	20.4 (16.0-30.0)	18.0 (14.0-24.0)	21.0 (19.9–27.5)	29.5 (21.7-45.0)	22.5 (12.0-31.4)	.074^c^
**GPT U/L** **151 patients**	Median (IQR)	18.0 (12.0-31.0)	19.2 (13.0-32.0)	14.5 (10.8-24.0)	16.3 (14.5–23.1)	39.4 (13.9-47.0)	15.5 (11.1–30.5)	.075^c^
**Albumin g/dL** **143 patients**	<3.5 ≥3.5	41 (28.67) 102 (71.33)	16 (11.19) 64 (44.76)	16 (11.19) 23 (16.08)	3 (2.10) 7 (4.90)	3 (2.10) 4 (2.80)	3 (2.10) 4 (2.80)	.084^a^
**ESR mm/h** **141 patients**	<20 20-40 >40	59 (41.84) 38 (26.95) 44 (31.21)	35 (24.82) 18 (12.77) 27 (19.15)	11 (7.80) 13 (9.22) 15 (10.64)	6 (4.26) 2 (1.42) 0 (0.00)	4 (2.84) 3 (2.13) 1 (0.71)	3 (2.13) 2 (1.42) 1 (0.71)	.188^a^
**Coproculture ** **122 patients**	+	8 (6.56)	2 (1.64)	1 (0.82)	1 (0.82)	2 (1.64)	2 (1.64)	**.034^a^**
**Calprotectin mcg/g** **113 patients**	<50 50-149 150-499 500-1000 >1000	28 (24.78) 5 (4.42) 30 (26.55) 33 (29.20) 17 (15.04)	21 (18.58) 6 (5.31) 7 (6.19) 21 (18.58) 9 (7.56)	4 (3.54) 3 (2.65) 12 (10.62) 7 (6.19) 6 (5.31)	2 (1.77) 1 (0.88) 3 (2.65) 0 (0.00) 0 (0.00)	1 (1.88) 0 (0.00) 1 (0.88) 3 (2.65) 1 (0.88)	0 (0.00) 0 (0.00) 2 (1.77) 2 (1.77) 1 (0.88)	.06
**pANCA** **97 patients**	+	17 (17.53)	13 (13.40)	1 (1.03)	0 (0.00)	2 (2.06)	1 (1.03)	.175^a^
**ASCA** **89 patients**	+	12 (13.48)	2 (2.25)	8 (8.99)	1 (1.12)	0 (0.00)	1 (1.12)	**.005^a^**
**Clostridiodes difficile ** **88 patients**	+	4 (4.55)	0 (0.00)	1 (1.14)	2 (2.27)	0 (0.00)	1 (1.14)	**.042^a^**
**GGT U/L** **79 patients**	Median (IQR)	16 (9-28)	11.8 (8.0-21.5)	21.0 (12.0-37.2)	25.8 (15.6-36.0)	32.0 (24.5-38.0)	22 (22-22)	**.014** ^c^
**Film array** **50 patients**	+	4 (8.00)	2 (4.00)	2 (4.00)	0 (0.00)	0 (0.00)	0 (0.00)	.668^a^

a Fisher’s exact test.

b Platelets = thousands.

c Kruskal-Wallis test.

Abbreviations: g/dL, grams per deciliter; mcg/g, micrograms per gram; mg/dL, milligrams per deciliter; mm/h, millimeters per hour; IQR, interquartile range; ASCA, anti-Saccharomyces cerevisiae antibodies; pANCA, perinuclear anti-neutrophil cytoplasmic antibodies; GPT, glutamic–pyruvic transaminase; GOT, glutamic-oxaloacetic transaminase; ESR, erythrocyte sedimentation rate; GGT, gamma-glutamyl transferase; CRP, C-reactive protein.

#### Radiological findings

Ultrasound was performed in 101 patients (48.32%), and only 21 (20.79%) patients had abnormal findings which included thickening of the bowel wall in any segment of the intestine, nodes larger than 5 mm, hyperemia of the wall, and loss of haustration in the colon. Computed tomography was performed in 30 patients (14.35%), of which 16 (53.33%) had noted abnormalities including enhanced and thickened (>3 mm) bowel wall, striation and increased vascularization of mesenteric fat, congestion of the vasa recta (comb sign) and lymph nodes >1 cm 55 (26.31%). Among patients who underwent magnetic resonance enterography (MRE), the following findings were observed: bowel wall thickening >3 mm, predominantly involving the rectum (21.6%), sigmoid colon (32.4%), and left colon (24.3%). Small bowel wall thickening >2.5 mm predominantly affected the ileum. Colonic wall stratification was primarily observed in the cecum and at the ileocecal valve. Loss of haustration was most pronounced in the sigmoid colon, left colon, and rectum, while mesenteric fat stranding was most frequently noted in the rectum and sigmoid colon. Additionally, lymphadenopathy (>1 cm in diameter) was identified in various segments, with the highest prevalence in the sigmoid colon.

Bone densitometry was requested in a few cases only.

#### Endoscopic findings

Esophagogastroduodenoscopy was performed in 166 patients (79.42%), 93 (56%) of which had edema, erythema, erosions, friable mucosa, nodularity, and ulcers in the upper gastrointestinal tract.

All patients had a colonoscopy with findings that included edema, erythema, friability, superficial and deep ulcers, loss of vascular pattern, fibrin membranes, serpiginous ulcers, and strictures in the colon and terminal ileum. Based on colonoscopy findings, classification of severity was completed. PUCAI score was assessed in 50 patients diagnosed with UC with a median of 45, consistent with moderate severity (IQR 25-65), with a minimum value of 5 and a maximum of 85. PCDAI was recorded in 11 patients with CD with a median of 45, also consistent with moderate severity (IQR 35.5-57.5), with a minimum value of 30 and a maximum of 80.

At the end of follow-up, the PUCAI scores of 63 patients with TUC and AUC had a median of zero, consistent with remission, while the PCDAI of 23 patients with CD had a median of 10, also consistent with remission.

Cannulation of the ileum was achieved in only 75 of 209 patients (35.8%), of whom 30.6% had changes suggestive of CD.

Enteroscopy was completed in 7 patients (3.34%), 5 of which (71.43%) had findings consistent with CD with edema, aphthous ulcers, erosions and ulcers. One of the patients had 2 areas of short stenosis without complete occlusion of the lumen.

Five patients had small bowel capsule endoscopy, and two of these had findings suggestive of CD including edema, aphthous ulcers, exudates, erythema, and ulcers.

#### Histologic finding

Histology reports also showed notable variability. However, the histological features reported reinforced the diagnosis of IBD. The most commonly identified histopathological alteration was crypt distortion accompanied by inflammatory changes. This was followed by architectural disarray and edema with a mixed infiltrate of neutrophils and lymphocytes, consistent with both acute and chronic inflammation. Granulomas were observed in a smaller subset of cases, approximately 12%. Notably, significant eosinophilic infiltration was detected in 32% of the specimens.

### Treatment

#### Initial treatment

Of the 196 patients with reclassification data, initial treatment information was available in 184 cases. Initial treatment regimens were divided into 4 groups as detailed below:

**Regimen A:** 66 (35.86%)

- Oral mesalamine + oral prednisone/prednisolone
- Mesalamine oral + methylprednisolone IV
- Sulfasalazine
- Sulfasalazine + prednisone/prednisolone

**Regimen B:** 59 (32.00%)

- Azathioprine + mesalamine
- Methotrexate + mesalamine
- Azathioprine + prednisone/prednisolone
- Methotrexate + prednisone/prednisolone
- Azathioprine + mesalamine + prednisone/prednisolone
- Methotrexate + mesalamine + prednisone/prednisolone

**Regimen C**: 38 (20.65%)

- Infliximab
- Adalimumab
- Infliximab + azathioprine
- Adalimumab + azathioprine

**Regimen D:** 21 (11.41%)

- Partial enteral nutrition 9 (42.85%)
- Exclusive enteral nutrition 12 (57.14%)

Patients with CD were the only group managed under treatment regimen D (exclusive and partial enteral nutrition). In the total group of 184 patients, 54 patients (27.55%) received additional therapies alongside regimens A, B, C or D, including mesalamine enemas in 37 patients, probiotics in 12, oral budesonide in 3, and turmeric in 2.

#### Pharmacological maintenance treatment

Maintenance regimen data were documented for 135 patients. These regimens were grouped as outlined below.

**Regimen E:** 12 (8.88%)

- Mesalamine oral + prednisone/prednisolone
- Mesalamine oral + methylprednisolone IV
- Sulfasalazine
- Sulfasalazine + prednisone/prednisolone

**Regimen F:** 45 (33.33%)

- Azathioprine + mesalamine
- Methotrexate + mesalamine
- Azathioprine + prednisone/prednisolone
- Methotrexate + prednisone/prednisolone
- Azathioprine + mesalamine + prednisone/prednisolone
- Methotrexate + mesalamine + prednisone/prednisolone

**Regimen G:** 78 (57.7%)

- Infliximab
- Adalimumab
- Infliximab + azathioprine
- Adalimumab + azathioprine
- Infliximab + methotrexate

For 21 patients who received exclusive enteral nutrition (Regimen D) as their initial therapy, maintenance treatment data were available for 9. These individuals were included in one of the established pharmacological regimens.

Corticosteroids administration was predominantly oral. In severe UC cases or in relapses, intravenous presentations were used for less than 5 days, with progressive weaning in the course of the first month.

The selected maintenance regimens were continued until the end of the observation period.

Two of the patients that received biologics required transition to vedolizumab/ustekinumab due to loss of response with little data available on their progress.

#### Surgical interventions

Surgical procedures were necessary in a small subset of patients: total colectomies were performed in 2 patients with TUC and 1 with CD, small bowel resections and seton placement were required in 2 patients with CD.

### Adverse events

Corticosteroids: Of 98 patients who took corticosteroids, 12 patients (12.2%) had adverse reactions including alopecia and acne, Cushing’s syndrome, nephritis, osteoporosis, hypertension, cataracts and uveitis. No adverse events were recorded among patients treated with oral budesonide.

Mesalamine: Of 76 patients, 12 (15.8%) had headache, abdominal pain, fatigue, dizziness, syncopal episodes, vomiting, and nephritis.

Azathioprine: Of 58 patients, 2 (3.45%) developed hepatitis and pancreatitis.

Biologics: No adverse effects were reported in the 38 patients who received biologic therapies.

### Response to treatment

In the 184 patients, remission and relapse were correlated with initial treatment regimens. Deep remission was found in 65 (35.32%), defined and included in the REDCap form as: “absence of symptoms, normalization of laboratory parameters and mucosal healing,” of which 21 (32.31%) had additionally normalization of histology. Among the 65 patients, 10 (15.38%) began therapy with first-line medications, 18 (27.69%) with immunomodulators, and 18 (27.69%) with biologic agents.

One or more relapses occurred in 98 patients (53.26%), 22 (22.44%) initiated treatment with first-line medications, 26 (23.46%) with immunomodulators, 20 (20.40%) with biologic agents, and 30 (30.61%) with others treatments.

During the follow-up period, the death of two patients (0.96%) with CD was reported: one with infantile onset and the other with very early onset. The cause of death was not specified.

## Discussion

Epidemiological knowledge is essential to plan strategies for population health. In IBD, it is well known that genetic and environmental factors affect the clinical course and phenotype of the disease, and importantly as recent research suggest, differences in the microbiome play an essential role in the clinical course of these patients. These factors are obviously different between Latin American countries and countries in Europe and North America that have had a longer history of patients presenting with IBD. There are limited studies evaluating genetic differences between populations of European ancestry and Latin American populations but studies completed in Puerto Rico and Florida have highlighted some similarities in risk loci shared between IBD patients of Hispanic and European ancestry. However, these genetic studies are not applicable to all of Latin America, given the diversity of the population, which also is present in a smaller scale in Colombia given our different backgrounds and migratory patterns.^16^ The South American MicroBiome Archive revealed unique diversity and between-population uniqueness in the continent, and ample differences with the microbiome studies reported in Europe and North America.^17^ These variations likely have an impact in the incidence and clinical characteristics of our pediatric patients with IBD.

This is the first registry of this kind that has been established in Colombia and one of the few that has been conducted in any country in Latin America, highlighting differences and similarities with other populations. There is limited literature that contemplates the classification by age of onset of IBD and with the criteria of PIBD-classes in our population,^18^^,^^19^ and the introduction of these reclassifications in this registry, unified the criteria with up-to-date concepts despite the inherent risk of bias when including retrospective data for more than 20 years.

Despite retrospective and prospective directionality of the collection of data, the introduction of the current classification based on the criteria of the IBD-classes^12^^,^^13^ and division into groups according to age of onset of the disease,^10^^,^^11^ allowed control of the information biases with the strategies implemented. Additionally, by carefully implementing a unified classification criterion throughout the country’s pediatric care centers, a better understanding of IBD and the unification of diagnostic criteria was made possible.

No marked differences were found by gender. The finding of one patient with neonatal onset should alert healthcare providers about the possibility of IBD presenting at a young age even in countries where the prevalence and incidence of IBD is lower than what has been described in the literature of North American, European, and Asian countries. The presence of patients with very early onset and early onset in our population, also highlights this trend similar to the global one but with less frequency than in countries with historically higher rates of IBD.^20^ This observation raises concerns about emerging environmental factors in Latin America that may influence epigenetic regulation and microbiome composition, thereby contributing to increase the onset of IBD. These factors include changes in diet, hygiene changes, exposure to antibiotics, proton pump inhibitors, and increased environmental toxins such as air pollution, which have emerged over the last few decades and have shown variable associations with pediatric IBD incidence.^21^

The higher frequency of UC over CD has remained constant in adults^9^^,^^22^; however, in pediatric populations, the epidemiological behavior is variable and dependent on geographical area. In South America and the Caribbean, the difference in incidence between UC and CD has not changed and mimics the behavior in adults.^22^ In Colombia, when differentiating UC (TUC+AUC) (64.6%) from CD (CD+CCD) (32.1%), the proportion remains similar to previous findings reported from an academic center in Bogota in 2012, as well as with anecdotal unpublished observations from other centers (personal communication).

Regarding demographic differences, IBD predominates in urban areas, which could be related to environmental exposure to pollutants^23^ and higher consumption of ultra-processed foods^24^ that can alter the gut microbiome, leading to the onset or perpetuation of inflammatory mucosal damage.^25^

This pattern is evident in our data. Also, patients in rural areas in Colombia are exposed to parasitic infections early in life and often experience poorer hygienic conditions, which have been shown to impact immune response in these patients since early infancy. These factors could contribute to the lower prevalence of IBD observed in our rural population. Colombia has highly diverse geography encompassing all climatic zones and is divided into 6 natural regions: Andean, Caribbean, Pacific, Orinoquia, Amazon, and Insular. The highest population density is concentrated along the 3 mountain ranges of the Andean region. Considering the same environmental criterion, the concentration of patients in the Andean area of Colombia’s geography is notably higher than in other areas. This could be expected since these are more populated areas, but, it is striking that the incidence of IBD is much higher in provinces that share altitude and agricultural activities such as coffee cultivation, an observation that is replicated in Colombian adult patients.^7^ Comparable findings in coffee-growing areas with similar characteristics have not been described. Among the 32 provinces comprising the national territory, no IBD cases were identified in the Amazon or Orinoquia regions, both characterized by rainforest ecosystems. While these regions have the lowest population density and the least access to healthcare services, it is possible that these findings are due to lower diagnostic rates and environmental factors that directly impact the microbiome composition, and lower presence of risk loci of European ancestry given the high indigenous population in this regions.

Socioeconomic status can also impact disease history, and this is reflected in our registry where 83% of patients were concentrated in the 3 lowest socioeconomic strata, a difference that was statistically significant. This finding contrasts with the previous concept that IBD was primarily a disease of affluent populations, based on data from Europe, Israel, and North America, and correlated with the hygiene hypothesis.^26^^,^^27^ It is important to note that lower socioeconomic status has been studied and associated with poorer outcomes and reduced access to care and treatment. In our registry, data regarding access to care, medication use, and clinical outcomes were not stratified by socioeconomic status, which leads to an interesting point that should be further studied.^28^^,^^29^

It is encouraging to find that IBD care was not exclusive to academic or large medical centers indicating the growing interest and awareness on IBD among Colombian pediatric gastroenterologists. Gastroenterologists that lack this academic support have managed the care of these patients by reaching out to more experienced centers via internet communications, interactive non-face-to-face meetings or by chats, which allow another modality of interdisciplinary work, a strategy that has proven to be remarkably useful.

In our registry, the time elapsed from the onset of symptoms to diagnosis was a median of 6.6 months but in some patients it took years to establish the diagnosis. This is similar to previous studies that report the median duration of symptoms before diagnosis to be 2-12 months for CD and 2-7 months for UC. However, approximately 25% of patients have to wait up to 5 years from the onset of symptoms to obtain a final diagnosis of IBD, despite being seen by a doctor or despite having one or more visits to the emergency room.^30^ Particularly in our Colombian population, possible factors that could have impacted this include the socioeconomic and logistic factors that could limit timely access to health services.^31^ In addition to these, the high prevalence of infectious pathology that presents with similar symptoms, may have led to attributing symptoms to infectious and allergic etiologies^32^; and transient improvement with dietary management could also have contributed to diagnostic delays. Furthermore, the previous absence of diagnosis of IBD in our country has likely made it a less frequently considered diagnosis, particularly among physicians trained more than two decades ago. In contrast, the time elapsed from diagnosis to the start of treatment (median of 11.2 days) was similar to or even shorter than that reported in the literature.^33^

The clinical characteristics observed in our population correlate with commonly reported symptoms of abdominal pain, bloody diarrhea, and weight loss. Almost 50% of our patients with TUC presented these symptoms, as opposed to only 28% of the patients with CD, highlighting the need to consider additional signs and symptoms for IBD diagnosis. In UC, nocturnal diarrhea was also a frequent symptom, and in CD, perianal manifestations including skin tags and anal fissures were present in a considerable percentage of patients^34^ similar to reports in the literature in other populations.

Even though we did not study the onset of EIM prior to IBD symptoms, it is worth mentioning that these can precede IBD gastrointestinal symptoms in 25%-30% of patients.^1^^,^^35^ In this registry, 86 patients (43.9%) had EIM with growth failure outweighing axial and peripheral arthropathies. The inclusion of atypical systems beyond the classical joints, skin, liver, and eyes,^36^ likely may have contributed to the increased prevalence of EIMs observed in our cohort, because patient enrollment began in 1999, when standardized criteria for defining and identifying EIMs, particularly those involving atypical systems, were not well-established.^37^ It is noteworthy that only 25.56% of patients had anemia (Hb <10 g/dL), even though IBD is often associated with anemia without us having a clear explanation.

The low reporting of ASCA and antineutrophil cytoplasmic antibodies (ANCA) could be a reflection of their limited use. The findings in fecal matter should be highlighted: the presence of calprotectin levels above 150 mcg/dl in 70% of patients confirms its importance.

The low positivity of stool cultures and the very low positivity of Clostridiiodes difficile compared to other series should be the subject of analysis.

The radiological findings were diverse, but the request for ultrasound and MRI, with special protocols, as studies of choice in the diagnostic approach, demonstrates a relative uniformity of criteria for their use. We do not know if densitometry was not reported or was not requested, but it is striking that it is not taken into account as a valuable examination for the comprehensive management of the patient and highlights the need to standardize the health maintenance care for our patients. Finally, the low number of enteroscopy and capsule endoscopy reports is likely related to limited access in non-tertiary care centers. This finding should encourage efforts to promote their use in specific cases and to improve availability for patients managed in private practices or smaller institutions.

Endoscopic findings were heterogeneous and not always useful in the diagnosis,^13^ suggesting the need for standardized strategies in training and reporting, based on the phenotypes of the PIBD-classes.^38^ Likewise, the remarkable variability of the descriptions in colonoscopy should lead us to the systematic use of the severity scoring indices for endoscopic findings.^39–41^ The percentage of ileocecal valve cannulation lower than the standards raises, the need to evaluate the reasons for this discrepancy including variations in personal and institutional practice and training, and prompt interest to design strategies to close this gap between the reported percentage within our population and the percentages reported in the literature.^42^

Disparities in histological reports were possibly due to the lack of familiarity and experience of general pathologists in Colombia with the histological characteristics of pediatric IBD, its phenotypes and diagnosis as opposed to other countries with higher incidence of IBD. Nevertheless, histology interpreted in conjunction with clinical, laboratory, and imaging findings, they completed and confirmed the diagnosis of IBD in our patients.

Given the variability of medications and interventions for management, we grouped them into regimens that would facilitate a reasonable evaluation of management and outcomes. There were 4 regimens for initial therapy and 3 for maintenance. For initial treatment, the most commonly used initial regimen was mesalamine or sulfasalazine with or without oral or parenteral corticosteroids. Topical sulfasalazine and mesalamine, as well as mesalamine suppositories or foam were not used often despite the advantages reported in the literature. In our data, azathioprine was the only immunomodulator used in combination with mesalamine, corticosteroids and/or well anti-TNFs. Methotrexate was not used, despite being an alternative to azathioprine, even though there were two serious adverse events (pancreatitis and hepatitis) related to its use.^43^

Anti-TNFs as initial treatment of IBD in pediatrics have been increasingly used. In this registry, biologics were utilized only in 20.65% of patients for initial treatment, likely due to their limited availability in the initial years of the registry, but tripled to 57.7% for maintenance therapy. This increase in use of antiTNFs for maintenance can be associated with severity of the disease in our region, the increased familiarity with their use, poor response to the initially chosen therapy or with limited availability in the initial years of the registry. Infliximab was administered in some cases as monotherapy. Contrary to some recommendations for combination therapy, in our registry, there were no adverse events reported in 20 years of management of pediatric patients with IBD. This could have been due to underreporting or lack of documentation.

Enteral nutrition as a treatment strategy in pediatrics, has been shown to be effective in inducing remission in CD. It is interesting and valuable to find in our study that in the 63 patients diagnosed with CD and CCD, one third received exclusive or partial enteral nutrition despite being a novel treatment in our Colombian setting. Unfortunately, we had very little data on the compliance and response of patients to this treatment. A variety of formulas were used, possibly due to limited local availability or logistics.

When reviewing the data on maintenance therapies, only 8.8% of patients remained on the first-line regimen which reflects the limited efficacy of mesalamine and the need to consider treatment with immune modulators or biologics as initial therapy. It is possible that patients were initially treated with mesalamine and/or steroids due to difficult access to biologics or an underestimation of disease severity at the time of diagnosis, despite endoscopic scores in our population were consistent with moderate to severe disease in 69% of the patients with UC and 100% of the patients with CD. The 3-fold (20.6%-57.7%) increase in anti-TNFs (Infliximab/Adalimumab) used during maintenance therapy reflects the current standard treatments for these patients, especially those patients with VEO IBD.

In this cohort, clinical and endoscopic remission was achieved in 65 patients (31.1%), of whom 21% also attained histologic remission. These rates are far from where our goals of treatment success should be and underscore the need of standardized diagnosis, classification, and treatment of pediatric IBD patients in Colombia and Latin America. Deep remission, defined as “*absence of symptoms, normalization of blood biochemistry, and calprotectin and mucosal healing*,”^44^^,^^45^ is the basis of “treat to target.” Beyond this, achievement of histological normalization, which has been called deep and sustained or complete remission,^46^ can radically change the disease course.

With the rapidly increasing incidence of IBD in Latin America, raising awareness and improving education among all professionals involved in the care of these patients is essential. Efforts should focus on establishing clear distinctions between IBD and other conditions, such as allergic and eosinophilic gastrointestinal pathology, infectious processes, irritable bowel syndrome, and recurrent abdominal pain.^47–49^

The management options described in this registry stress the need for guidelines and education in the management of pediatric patients with IBD in Colombia, following a comprehensive approach and considering a differential analysis of the clinical presentations of IBD including the age of diagnosis. Early-onset IBD and very early-onset IBD are associated with greater severity, faster progression, and less favorable outcome, necessitating the use of more effective therapies. Furthermore, availability of treatments has improved markedly over the past years allowing for better treatment of this growing population.

## Conclusion

In conclusion, the information in this registry facilitates the understanding and unification of concepts for improved care of pediatric patients with IBD in Colombia. Differences between this cohort and reported pediatric series in other countries highlight the need for further studies to differentiate genetic and environmental factors that directly impact the clinical course of these patients. Also, consideration of local resources and logistics that directly determine the therapeutic options is essential in the care of these children. Moreover, efforts to enhance awareness of the increasing incidence of the disease and education on classifications and therapeutic options will have a direct impact in the health of these patients. These efforts should be aimed at families and caregivers, primary care providers including family doctors and pediatricians, and pediatric gastroenterologists throughout the country and including less populated and rural areas. The information compiled here will be the basis of future research in this population in Colombia that can be extrapolated and include other Latin American countries and populations.

## Supplementary Material

otag038_Supplementary_Data

## Data Availability

The database generated from REDCap with the available information of each of the 209 patients was exported to an Excel and then to Stata. The supplementary data are available at IBD Journal online in excel format. For additional information, please contact the corresponding author.

## Supplementary material

Supplementary material (IBD Colombian Register Database.xlxs) is available at *Crohn’s and Colitis 360* online.
